# Adaptogenic and nootropic activities of aqueous extract of *Vitis vinifera *(grape seed): an experimental study in rat model

**DOI:** 10.1186/1472-6882-5-1

**Published:** 2005-01-19

**Authors:** Satyanarayana Sreemantula, Srinivas Nammi, Rajabhanu Kolanukonda, Sushruta Koppula, Krishna M Boini

**Affiliations:** 1 Pharmacology Division, Department of Pharmaceutical Sciences Andhra University, Visakhapatnam – 530 003, Andhra Pradesh, India; 2 Department of Physiology, University of Tübingen, D 72076 Tübingen, Germany

## Abstract

**Background:**

The aerial parts of *Vitis vinifera *(common grape or European grape) have been widely used in Ayurveda to treat a variety of common and stress related disorders. In the present investigation, the seed extract of *V. vinifera *was evaluated for antistress activity in normal and stress induced rats. Furthermore, the extract was studied for nootropic activity in rats and *in-vitro *antioxidant potential to correlate its antistress activity.

**Methods:**

For the evaluation of antistress activity, groups of rats (n = 6) were subjected to forced swim stress one hour after daily treatment of *V. vinifera *extract. Urinary vanillylmandelic acid (VMA) and ascorbic acid were selected as non-invasive biomarkers to assess the antistress activity. The 24 h urinary excretion of vanillylmandelic acid (VMA) and ascorbic acid were determined by spectrophotometric methods in all groups under normal and stressed conditions. The nootropic activity of the extract as determined from acquisition, retention and retrieval in rats was studied by conditioned avoidance response using Cook's pole climbing apparatus. The *in vitro *antioxidant activity was determined based on the ability of *V. vinifera *to scavenge hydroxyl radicals.

**Results:**

Daily administration of *V. vinifera *at doses of 100, 200 and 300 mg/kg body weight one hour prior to induction of stress inhibited the stress induced urinary biochemical changes in a dose dependent manner. However, no change in the urinary excretion of VMA and ascorbic acid was observed in normal animals at all the doses studied. The cognition, as determined by the acquisition, retention and recovery in rats was observed to be dose dependent. The extract also produced significant inhibition of hydroxyl radicals in comparison to ascorbic acid in a dose dependent manner.

**Conclusion:**

The present study provides scientific support for the antistress (adaptogenic), antioxidant and nootropic activities of *V. vinifera *seed extract and substantiate the traditional claims for the usage of grape fruits and seeds in stress induced disorders.

## Background

Stress can be described as the sum total of all the reactions of the body, which disturb the normal physiological condition and result in a state of threatened homeostasis. Stress is an internationally recognized phenomenon fortified by advancement of industrialization in a demanding civilization. Thus, every individual is likely to face stressful situations in day-to-day life. Stress represents a reaction of the body to a stimulus that tends to alter its normal physiological equilibrium or homeostasis and has been defined as a nonspecific response of the body to any demand imposed on it [1]. Since the introduction of adaptogens, several plants have been investigated, which were once used as tonics due to their adaptogenic and rejuvenating properties in traditional medicine [2]. The drugs of plant origin are gaining increasing popularity and are being investigated for remedies of a number of disorders including antistress (adaptogenic) activity [3]. The initial studies on *Ocimum sanctum *[4], *Withania somnifera *[5] opened a vast area of research and substantial work has been carried out on plants such as *Eleuthrococcus senticosus *and *Panax ginseng *[6].

*Vitis vinifera *(Linn.) (Family: Vitaceae) also called as common grape or wine grape or European grape is one of the fruit crops most widely grown throughout the world [7]. In the indigenous Indian system of medicine (Ayurveda), the aerial parts of *V. vinifera *have been widely used to treat a variety of common and stress related disorders [8]. The composition and properties of grape seeds have been extensively investigated, and reported to have many favourable effects on human health such as the lowering of low-density lipoprotein [9,10], reduction of cardiovascular diseases and cancer [11]. In addition, the seed extracts of *V. vinifera *are reported to have antimicrobial and free radical scavenging properties [12]. In the present investigation, the antistress activity of *Vitis vinifera *was evaluated *in-vivo*, in normal and stress induced rats following a biochemical approach. The antioxidant potential of the extract was evaluated *in-vitro *to support the antistress activity. The plant extract was further evaluated for nootropic activity using conditioned avoidance response in rats.

## Methods

### Preparation of extract

The riped fruits of *Vitis vinifera *were collected from the Chittor district of Andhra Pradesh, India in the month of October and the seeds were separated from the pulp and shade dried. The dried powdered seed material of *Vitis vinifera *(5 kg) was extracted with boiling water (25 L) for 45 minutes and the filtrate was evaporated under vacuum below 70°C in a vacuum drier to give a final yield of 50 gm.

### Chemicals used

Vanillylmandelic acid (VMA) and scopolamine butylbromide (SBB) were purchased from Sigma-Aldrich, St. Louis, USA, while ascorbic acid was purchased from Loba Chemie, Mumbai. All other reagents used were of analytical grade.

### Animals

All animal experiments were performed in accordance with our Institutional Animal Ethics Committee and by the animal regulatory body of the government (Regd. No. 516/01/A/CPCSEA). Albino rats of either sex obtained from Ghosh Enterprises, Kolkata were used in the study. They were housed six per cage at a temperature of 22 ± 2°C with 12 h light/ dark cycle under controlled environment. Rats were fed with standard pellet diet (Ratan Brothers, Hyderabad), and water *ad libitum*. Animals were kept for seven days in laboratory for habituation.

### Antistress activity

Rats of either sex weighing between 120–150 gm were divided into four groups (I, II, III, IV) each containing six animals. The 24 h urine sample from each group was collected into two different beakers, one containing 5 ml of 10% oxalic acid for the spectrophotometric determination of ascorbic acid at 550 nm [13] and the other containing 0.5 ml of 6 *N *hydrochloric acid for the determination of vanillylmandellic acid (VMA) spectrophotometrically at 360 nm [14]. The experimental protocol was divided into four phases. In the first phase of the experiment, 24 h urine samples were collected in all the four groups and subjected to analysis for both VMA and ascorbic acid and the normal values were recorded for seven consecutive days. In the second phase, the animals in each group were subjected to fresh water swimming stress individually. In this method, rats were forced to swim until exhausted (three to four minutes) in a cylindrical vessel of 60 cm height and 45 cm diameter containing water at room temperature (28°C). Water depth was always maintained at 40 cm. The 24 h urinary levels of VMA and ascorbic acid under stressed conditions were determined again as described above daily for seven consecutive days. The third phase of the experiment consists of administration of *V. vinifera *extract to the same groups of animals after having recovered completely to normal condition. Groups II, III and IV were administered orally with *V. vinifera *(suspended in 2% gum acacia) at daily doses of 100, 200 and 300 mg/kg body weight respectively for seven consecutive days while group I serving as control. The 24 h urine samples were collected and the levels of both VMA and ascorbic acid were determined. The final phase of the experiment consisted of administration of *V. vinifera *extract to the same groups of animals after a recovery period of one week. Groups II, III and IV were administered orally with *V. vinifera *at doses of 100, 200 and 300 mg/kg body weight respectively, one hour prior to the daily induction of stress for seven consecutive days while group I serving as control. The 24 h urine samples were collected and analyzed for VMA and ascorbic acid for seven consecutive days to study the influence of the extract on the stress induced biochemical changes.

### Nootropic activity

The Nootropic activity of *V. vinifera *was evaluated by using the conditioned avoidance response (*CAR*) in rats as described by Cook and Weidley [15]. Rats were divided into 4 groups each containing six animals. Groups II, III and IV were administered orally with 100, 200 and 300 mg/kg body weight respectively of *V. vinifera *(suspended in 2% gum acacia) while animals in group I were served as control. After 60 minutes, all the animals were subjected to a training schedule individually by placing inside the perspex chamber of the apparatus. After an accustomed period of five minutes to the chamber, a buzzer was given followed by a shock through the grid floor. The rat had to jump on the pole to avoid foot shock. Jumping on the pole functionally terminates the shock and this was classified as an escape while such jumping prior to the onset of the shock was considered as avoidance. The session was terminated after completion of 60 trials with an interval of 20–30 seconds given for each trial. This procedure was repeated at 24 h intervals until all groups reach 95 to 99% avoidance. After attaining complete training of a particular group, the animals were treated with a single dose of scopolamine butyl bromide (1 mg/kg body weight, i.p.), thirty minutes before the next day dosing. The training schedule was continued further with the daily doses of the extract and vehicle until they returned to normal level from scopolamine induced amnesia.

### Antioxidant activity

The antioxidant activity of *V. vinifera *was determined based on its ability to scavenge the hydroxyl radicals [16]. Hydroxyl radical scavenging activity was measured by studying the competition between deoxyribose and the extract for hydroxyl radicals generated from the Fe^3+^-ascorbate-EDTA-H_2_O_2 _system. The hydroxyl radicals attacks deoxyribose and eventually results in the formation of thiobarbituric acid reacting substances (TBARS). The reaction mixture containing deoxyribose (2.8 mM), ferric chloride (0.1 mM), EDTA (0.1 mM), H_2_O_2 _(1 mM), ascorbate (0.1 mM) phosphate buffer (20 mM, pH 7.4) and various quantities of the extracts in a final volume of 1 mL was incubated for 1 h at 37°C. Deoxyribose degradation was measured as TBARS by the method of Ohkawa et al., 1979 and the percentage free radical inhibition was calculated from control.

### Data and statistical analysis

The results are expressed as means ± standard error of means. Statistical analysis was done using Student's paired *t*-test. In all the cases, p < 0.05 was considered statistically significant.

## Results

### Antistress activity

**Figure 1:**
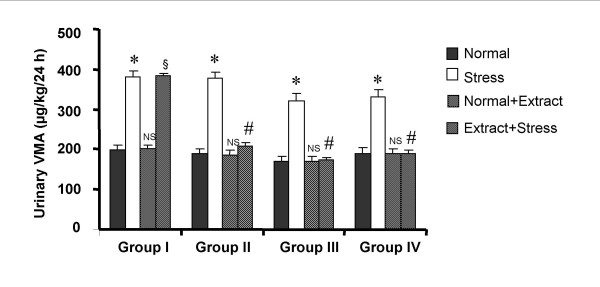
**Influence of *Vitis vinifera *seed extract on the 24 h urinary levels of VMA in normal and stress induced rats**. Each bar indicates the mean excretion of six animals. Significant difference from normal condition of the corresponding groups: *p < 0.05 Significant difference from stress condition of the corresponding groups: *#*p < 0.05 NS- No significant difference from normal condition of the corresponding groups. §No significant difference from stress condition.

**Figure 2:**
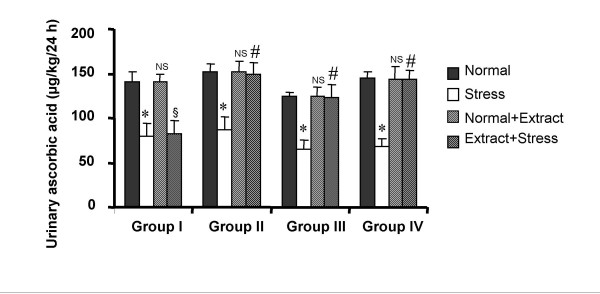
**Effect of *Vitis vinifera *seed extract on the 24 h urinary levels of ascorbic acid in normal and stress induced rats**. Each bar indicates the mean excretion of six animals. Significant difference from normal condition of the corresponding groups: *p < 0.05 Significant difference from stress condition of the corresponding groups: *#*p < 0.05 NS- No significant difference from normal condition of the corresponding groups. §No significant difference from stress condition.

The urinary data of VMA and ascorbic acid observed in various phases of the experiment are shown in Fig. 1 and Fig. 2 respectively. Induction of forced swim stress to the animals produced a significant increase in VMA and decrease in ascorbic acid excretion compared to their respective basal excretion in normal condition. Both the parameters were found to return to their normal levels in three to four days after withdrawal of stress. Daily treatment of *V. vinifera *to the animals under normal condition produced no change in the excretion of VMA and ascorbic acid compared to normal basal levels indicating that *V. vinifera *did not alter excretion of VMA and ascorbic acid in normal condition. Daily administration of *V. vinifera *one hour prior to the induction of stress inhibited the increase in VMA and decrease in ascorbic acid excretion which was manifested during stress alone. The inhibition was found to be significant at all dose levels in a dose dependent manner.

### Nootropic activity

**Figure 3:**
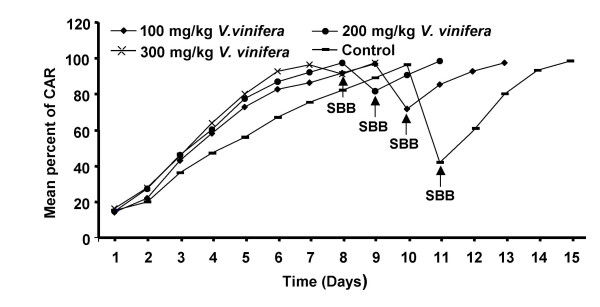
**Effect of *Vitis vinifera *seed extract on the mean percent of conditioned avoidance response after oral administration in rats**. Scopolamine butylbromide (SBB) was administered thirty minutes before the next day dosing with the extract after attaining complete acquisition.

The *CAR *of rats administered with the extract of *Vitis vinifera *or vehicle increased gradually to 95% over seven to ten days. The percent avoidance was always higher in the extract treated groups compared to vehicle treated control group. The acquisition (time to achieve 95% *CAR*) for the extract treated groups was quicker and found to be dose dependent. Animals receiving 300 mg/kg body weight of the extract have taken seven days whereas, groups treated with 200 and 100 mg/ kg doses of the extract required eight and nine days respectively to reach the point of acquisition (Fig. 3). Administration of scopolamine produced amnesia as seen from reduction in the observed CAR. However, continued treatment of *V. vinifera *produced better retention and recovery in a dose dependent manner than the vehicle treated animals.

### Antioxidant activity

**Figure 4:**
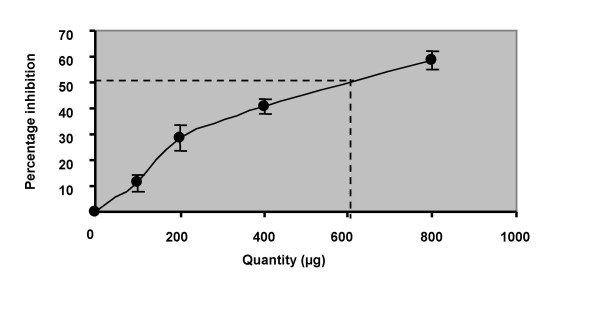
**Hydroxyl radical scavenging activity of *Vitis vinifera *in *in-vitro *systems**. Graphical representation of the concentration of *Vitis vinifera *required to inhibit 50 percent of hydroxyl radicals. Each point represents the mean percentage inhibition of six experiments.

**Figure 5:**
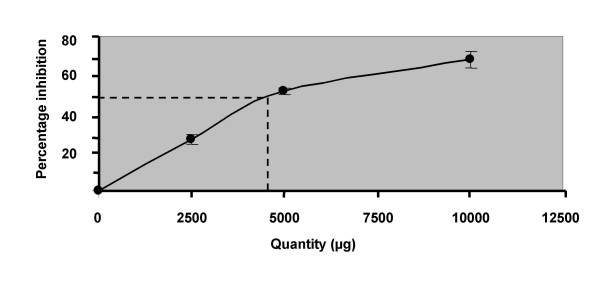
**Hydroxyl radical scavenging activity of ascorbic acid in *in-vitro *systems**. Graphical representation of the concentration of ascorbic acid required to inhibit 50 percent of hydroxyl radicals. Each point represents the mean percentage inhibition of six experiments.

Degradation of deoxyribose mediated by hydroxyl radicals generated by Fe^3+^/ascorbate/EDTA/H_2_O_2 _system was found to be inhibited by *Vitis vinifera*. The extract at quantities of 100, 200, 400 and 800 μg levels scavenged the hydroxyl radicals in a dose dependent manner. Ascorbic acid at concentration of 2500, 5000 and 10000 μg was also found to produce dose dependent inhibition of hydroxyl radicals. The quantity of the extract needed for 50% inhibition was found to be 610 μg (Fig. 4). Similar effect was produced by ascorbic acid at a concentration of 4875 μg. (Fig. 5).

## Discussion

Advancement in the understanding of processes leading to explore the reason for stress induced disorders cannot obscure the simple fact that the exhaustion of energy supply still forms the basis that triggers the disorders and collapse of energy metabolism following glucose deprivation in circulation [17]. The desire to control the coping mechanism has led to emergence of science of adaptation, focusing to elucidate the mechanism that may help in modification so that insufficient, excessive and unnecessary responses can be prevented.

Literature reports indicate that noradrenaline is released during stressful conditions [18] and metabolized to vanillyl mandelic acid(VMA) peripherally and 3-methoxy 4-hydroxyphenyl glycol (MOPEG) centrally. In the light of such reports, VMA, the major metabolite of sympathetic amines, was taken as indirect biochemical index to represent the increase in peripheral sympathetic activity during stress. In the present study, the increase in the urinary VMA excretion during stress was used as a non-invasive biochemical marker to study the antistress activity of *V. vinifera*.

Several studies also indicated that the tissue levels of ascorbic acid decreased on application of stress [19]. Ascorbic acid being a free radical scavenger [20], it is more likely utilized in scavenging the free radicals involved in stress resulting in its decreased urinary concentration and also it has role in the biosynthesis of noradrenaline [21]*i.e*., as a cofactor in the conversion of dopamine to noradrenaline [22]. Based on the above studies ascorbic acid excretion in urine was taken as an indirect biochemical index to indicate the influence of stress on catecholamine synthesis in rats and antistress (adaptogenic) activity of the *Vitis vinifera *extract on prior administration of stress induction.

Treatment with *Vitis vinifera *extract along with stress reversed the stress induced biochemical changes i.e., increase in urinary VMA levels and decrease in urinary ascorbic acid levels, in a dose dependent manner. A number of Indian medicinal plants like *Ocimum sanctum*, *Withania somnifera*, *Panax ginseng *etc have been identified for their antistress activity. It was concluded that the antioxidant activity of these plants was partly responsible for their antistress activity [23]. Based on these reports the antioxidant activity of *Vitis vinifera *extract was also done using hydroxyl radical assay method. It was found that *Vitis vinifera *extract has significant good antioxidant activity which was 8 fold more than that of ascorbic acid.

The antistress and antioxidant activities were correlated with the nootropic activity of the extract since the role of stress and free radicals have been implicated in the loss of memory, concentration and also in Alzheimer's disease [24,25]. The process of nootropic activity involves acquisition, retention and retrieval and is measured using conditioned avoidance response. The acquisition was quicker in the extract treated rats (100, 200, 300 mg/kg body weight) in comparision to control, indicating the involvement of antistress activity of the extract. When challenged with scopolamine butylbromide (1 mg/kg body weight), the amnesia was less in treated group showing better retention and recovery than control group and the *Vitis vinifera *extract was shown to decrease memory loss which could be due to its central cholinomimetic activitiy apart from its free radical scavenging mechanisms. Furthermore, the antioxidant activity of the seed extract provide mechanistic basis in relieving stress by way of combating oxidative damage.

## Conclusion

In conclusion, the present study provides scientific support for the antistress (adaptogenic), antioxidant and nootropic activities of *V. vinifera *seed extract and substantiate the traditional claims for the usage of grape fruits and seeds in stress induced disorders. Further investigations are required to characterize the active constituent(s) responsible for observed activities of the seed extract.

## Abbreviations

VMA: Vanillylmandelic acid

CAR: Conditioned avoidance response

SBB: Scopolamine butylbromide

EDTA: Ethylene diamine tetra acetic acid

H_2_O_2_: Hydrogen peroxide

## Competing interests

The author(s) declare that they have no competing interests.

## Authors' contributions

SS conceived the study, made substantial contributions in data analysis, data interpretation, writing of the manuscript and in coordination of the experiments. SN made substantial contributions in conceptualization of statistical analyses, drafting the final manuscript and designing the illustrations. RK and SK helped to conceptualize the work. KMB made significant contribution in designing the studies, conducting the experiments, interpretation of the data, and drafting the final manuscript. All authors read and approved the final manuscript.

## Pre-publication history

The pre-publication history for this paper can be accessed here:


